# Using the Intervention Mapping protocol to develop a family-based intervention for improving lifestyle habits among overweight and obese children: study protocol for a quasi-experimental trial

**DOI:** 10.1186/s12889-016-3766-6

**Published:** 2016-10-18

**Authors:** Tonje Holte Stea, Tommy Haugen, Sveinung Berntsen, Vigdis Guttormsen, Nina Cecilie Øverby, Kristin Haraldstad, Eivind Meland, Eirik Abildsnes

**Affiliations:** 1 Department of Public Health, Sport and Nutrition, University of Agder, Kristiansand, Norway; 2 Department of Health and Nursing Sciences, University of Agder, Kristiansand, Norway; 3 Department of Global Public Health and Primary Care, University of Bergen, Bergen, Norway

**Keywords:** Childhood obesity, Intervention Mapping protocol, Energy balance related behavior, Parental involvement

## Abstract

**Background:**

In light of the high prevalence of childhood overweight and obesity, there is a need of developing effective prevention programs to address the rising prevalence and the concomitant health consequences. The main aim of the present study is to systematically develop and implement a tailored family-based intervention for improving lifestyle habits among overweight and obese children, aged 6–10 years old, enhancing parental self-efficacy, family engagement and parent-child interaction. A subsidiary aim of the intervention study is to reduce the prevalence of overweight and obesity among those participating in the intervention study.

**Methods/design:**

The Intervention Mapping protocol was used to develop a tailored family-based intervention for improving lifestyle habits among overweight and obese children. In order to gather information on local opportunities and barriers, interviews with key stakeholders and a 1-year pilot study was conducted. The main study has used a quasi-experimental controlled design. Locally based Healthy Life Centers and Public Health Clinics are responsible for recruiting families and conducting the intervention. The effect of the study will be measured both at completion of the 6 months intervention study and 6 and 18 months after the intervention period.

An ecological approach was used as a basis for developing the intervention. The behavioral models and educational strategies include individual family counselling meetings, workshops focusing on regulation of family life, nutrition courses, and physical activity groups providing tailored information and practical learning sessions. Parents will be educated on how to use these strategies at home, to further support their children in improving their behaviors.

**Discussion:**

A systematic and evidence-based approach was used for development of this family-based intervention study targeting overweight and obese children, 6–10 years old. This program, if feasible and effective, may be adjusted to local contexts and implemented in all municipal health care institutions in Norway.

**Trial registration:**

NCT02247219. Prospectively registered on October 26, 2014.

## Background

The prevalence of childhood overweight and obesity in Europe has increased dramatically during the past decades [1]. A study from 2010 showed that 17 % of Norwegian children aged 6–11 years were overweight or obese [2]. Recently published data from the National Growth study have shown a stabilization of overweight and obesity in Norwegian third graders between 2008 and 2015 and in 2015, 17 % girls and 13 % boys were identified as overweight or obese [3].

Recent reforms in the Norwegian health care system places more responsibility on municipal health care institutions with regard to prevention and early intervention in the course of a disease [4]. This includes a focus on prevention of overweight and obesity among all age groups, including children [5]. In order to conduct screening, monitoring, prevention and management of childhood overweight and obesity, national guidelines on measuring children’s height and weight, prevention and management have been provided to public health clinics [6, 7].

Associations between overweight/obesity and body image dissatisfaction, low self-esteem, poorer self-assessed health status, potential social isolation and decreased quality of life have been documented among youth [8–10]. Other possible health consequences of early onset of obesity are muscular-skeletal, orthopedic and neurological alignments [11, 12]. In addition, substantial evidence supports the association between childhood overweight and obesity and high blood glucose levels and abnormal blood lipid levels, which can lead to premature cardiovascular disease, some types of cancer and diabetes [13, 14]. Although the prevalence of child obesity may be stabilizing in Norway and some other European countries, the prevalence is still high [15] and socioeconomic differences persists [16, 17]. Furthermore, several studies have demonstrated that overweight and obesity often persist and even increases into adulthood [18–20]. Thus, childhood obesity has both short term and long term impacts on health and well-being of individuals and yields a considerably financial cost to healthcare systems [21, 22]. However, the link between obesity and reduced health among children is not one way causal, but complex. The Director-General of WHO, Margaret Chan has stated “Childhood obesity is not just the result of lifestyle choices. It is driven by trans-generational, biological and environmental issues beyond the child’s control” [23]. Increased sedentary time and reduced daily physical activity in addition to unfavorable dietary habits resulting in positive increments in energy balance have been postulated as the main explanations for the pediatric obesity epidemic [24, 25].

According to the ecological model for health promotion, it is necessary to acknowledge the influence of both individual and environmental factors, including parent-child interaction, when planning health promotion interventions [26]. Although a literature review has confirmed that parents have a crucial role in shaping children’s dietary and physical activity behaviors and management of weight-related problems [27], only few studies promoting healthy sustainable life style changes in overweight and obese children have included parents. Further, Davison et al. [28] has emphasized the importance of, not only including family members as target groups, but invite them to contribute in the development, implementation and evaluation of childhood obesity prevention programs.

A recently published systematic research review concluded that complex lifestyle interventions involving structured strategies for change in diet and physical activity may reduce BMI and BMI z-score compared with less complex interventions [29]. However, few of the complex intervention studies identified in this review reported long-term effects [29], and studies that include a family component seldom focus on sustainable change in family behavior [28]. Therefore, national and international health organizations [5, 7] have called for structured and systematic development of family-based intervention studies that aim to reduce overweight and to prevent childhood obesity.

Intervention Mapping (IM) has been suggested as a useful and effective tool to further improve the development and application of theories to promote nutrition and physical activity (PA) [30]. Thus, the main aim of the present study is to use the IM process to systematically develop and evaluate a sustainable, tailored intervention program for improving lifestyle habits and enhance quality of life among overweight and obese children, aged 6–10 years old, and enhance parental self-efficacy, family engagement and parent-child interaction. A subsidiary aim of the intervention study is to reduce the prevalence of overweight and obesity among children participating in the intervention study.

## Methods

A multidisciplinary team of researchers with extensive experience in conducting clinical intervention studies has worked to realize this project according to a systematic stepwise approach that combines the use of the PRECEDE-PROCEED model and IM protocol [31]. The PRECEDE-PROCEED model is a conceptual model serving the needs of the present project with an educational and ecological approach in health program planning. IM is used as a planning approach to develop theory- and evidence based health promoting intervention programs. The IM process consists of six different steps; 1) performing needs assessment, 2) developing program objectives, 3) selection of intervention methods and practical strategies, 4) producing program components and materials, 5) plan for adoption and implementation and, 6) designing an evaluation plan. A benefit of IM is its linkage with community-based participatory research as it includes engagement of community members to identify and refine priority areas. Thus, the process of including IM in developing the present intervention program has secured that key stakeholders have influenced the development of the intervention program, and that the intervention has been tailored to fit local needs and available resources and competence.

### Step 1: Needs assessment

In the initial steps of the IM process, literature was reviewed and studies reporting effective intervention components in the prevention of childhood obesity were analyzed. In addition, we explored the research field utilizing qualitative research methods, including focus groups and in-depth interviews with parents and key stakeholders. These processes provided information about important behavioral and environmental risk factors and determinants of risk behaviors related to childhood obesity. Finally, a pilot study was conducted in order to gather information on local opportunities and barriers when targeting overweight children and their parents. As a result of the needs assessment, specific program objectives and outcomes were stated.

#### Target group

The needs assessment identified the following target groups; overweight/ obese children (iso-BMI ≥ 25) between 6 and 10 years old, their parents, public health nurses and other health care providers at Public Health Clinics (PHCs) and Healthy Life Centres (HLCs). HLCs are municipal health services offering individual- and group-based behavioral change programs with special attention on the promotion of healthy dietary habits, increasing physical activity level and smoking cessation [32].

#### Focus group interview

In order to identify barriers and facilitators to lifestyle changes, we conducted a focus group interview with public health nurses (*n* = 5) having long-term experience with screening and follow-up of overweight/obese children and their parents. The focus-group interview was conducted by a trained research assistant using a semi-structured interview-guide. The interview was audio recorded and transcribed verbatim and analyzed using systematic text condensation [33].

#### In depth-interviews

Parents of overweight or obese children (*n* = 8) were interviewed, providing self-reported information about barriers and facilitators of lifestyle changes amongst their children. A semi-structured interview guide with both open questions and scales was used as a tool, and the interviews were conducted in the child clinic by two health-care professionals. One of the health care professionals conducted the interview and another was observing and writing complementary notes. All interviews were audio recorded, transcribed verbatim and analyzed using systematic text condensation [33].

#### Pilot study

A 1-year pilot study including 8 overweight/obese children and their parents was carried out over a period of 2 years in cooperation with one of the participating municipalities and the locally based PHC and HLC. The families participating in the pilot study were recruited based on assessment of overweight and obesity after standard measurement of children’s height and weight in preschool and 3rd grade. The pilot study included 3–8 individual consultations with health care providers and group-based physical activity sessions once a week with activity leaders, which were trained and mentored by professionals.

### Step 2: Performance objectives, determinants and change objectives

Based on the program objectives formulated in the needs assessment, different performance objectives were formulated at the individual level (children) and at the interpersonal level (parents/caregivers). These performance objectives stated what the participants had to do or how the environment had to be modified to accomplish the health related behaviors described in the program objectives. Conducting the literature review, interviews with key stakeholders, and the pilot study helped us to identify important and actuable behavioral and environmental determinants of physical activity, dietary habits and regulation of family life.

Finally, matrices of specific intervention objectives (i.e. change objectives) were created by crossing the determinants with the performance objectives. These change objectives were formed as action statements, describing what the participants or environmental agents had to do in order to accomplish the health-related behaviors described in the program objectives.

### Step 3: Methods and strategies

In the third step of the IM process, we identified theory-based methods most likely to influence changes in the selected determinants and under what conditions the methods are most likely to be effective. Next, the theory-based methods were translated into practical strategies that matched the change objectives from the matrices.

### Step 4: Program development

In the fourth step of the IM process, the information from all previous steps was combined and led to the development of different program components and materials. Most of the different program components and material were tested and revised based on feedback from the participants in the pilot study. Regulatory, demographic and cultural differences were taken into account to ensure feasibility of implementing the intervention in all participating PHCs/HLCs.

### Step 5: Adoption and implementation

The focus of Step 5 was program adoption and implementation, including consideration of program sustainability. This step of the protocol was supported and informed by the interviews of key stakeholders and experiences from the pilot study indicating that the intervention had to be flexible enough to deal with the variability in local circumstances between different PHCs/HLCs participating in the study.

Agreement about the locally based strategies was reached during face-to-face meetings with key personnel responsible for the program adoption and implementation in the different participating PHCs/HLCs.

In the months before starting the intervention, the research group discussed with the PHCs/HLCs how to recruit participants and apply the different program components. They were also instructed in detail about the program characteristics and how to use the different program components.

For successful implementation of the present intervention, our research group focused on safeguarding behavioral capability, necessary skills and self-efficacy and realistic outcome expectations of those responsible for implementation and sustainability of the program.

### Step 6: Evaluation

In the sixth step of the IM protocol, a plan to evaluate the effectiveness, in addition to process evaluation of the intervention study, was developed. As part of the process evaluation, in depth interviews of important stakeholders (*n* = 5) involved in the program in three different Norwegian municipalities were conducted, in which important enhancers and barriers related to the adoption and implementation process were identified. All interviews were recorded digitally, transcribed verbatim and analyzed using systematic text condensation [33].

## Results and discussion

### Step 1: Findings from the needs assessment

Needs assessment conducted in the present study included a review of relevant literature, results from focus group- and in depth-interviews with public health nurses and parents of overweight and obese children, and experiences from a pilot study. In order to continuously identify relevant knowledge concerning the needs, motivations, and behaviors of the target group, the intervention planning and needs assessment may, in accordance with Bartholomew and colleagues [31], be described as iterative rather than linear process.

#### Literature review

Longitudinal studies has shown that TV viewing and other sedentary behaviors in combination with unhealthy dietary habits such as high consumption of energy dense food and drinks constitute a significant risk factor for excessive weight gain in early childhood [34, 35]. A population based cross-sectional study among Pakistani primary school children has also shown that dietary behaviors, including breakfast skipping and intake of unhealthy food items, low levels of physical activity and a sedentary lifestyle were independent predictors of overweight and higher BMI [36]. Results from the ENERGY project indicated that engagement in more MVPA and less sedentary time was associated with a more favorable weight status among girls, but for boys, MVPA seemed most important for weight status, while sedentary time appeared to be less relevant [37]. Further, a recently published cross-sectional study showed that Moderate-to-vigorous intensity physical activity (MVPA), sleep and screen time were important lifestyle behaviors associated with overweight/obesity among children [38]. Another cross-sectional study has confirmed an association between short sleep duration and childhood overweight and that short sleep duration was strongly associated with more television viewing and computer use [39]. In addition, previous studies have identified increased BMI as a significant factor associated with poor health related quality of life (HRQoL), and studies have shown effects of multidisciplinary treatment on HRQoL. A 6- months multicomponent, school-initiated obesity intervention program including school- and family-based components, nutritional education, structured fun-type skill-learning and exercise training showed decreases in body mass index, body fat percentage and better HRQoL after the program [40].

As the link between obesity and reduced health among children is not one way causal, but complex, a systematic research reviews has concluded that complex lifestyle interventions involving structured strategies for change in diet and physical activity in combination with strategies facilitating family involvement may reduce BMI and BMI z-score compared to less complex interventions [29, 41]. Another systematic review reported that a reduction in screen time among parents may contribute to reducing screen time of their child and that parents’ encouragement and support may contribute to increasing their child’s PA [42]. This latter mentioned review also pointed out that different approaches, including improving parenting practices, parental self-efficacy or changing parenting style, may effectively contribute to increasing PA time and decreasing screen time in children [42]. Further, the results from a randomized clinical trial have shown both positive short- and long-term effects after using an adjunct motivational and an autonomy-enhancing delivery approach to behavioral pediatric obesity management [43]. A cluster-randomized controlled trial aiming to prevent overweight and obesity in 6-year-old Swedish children living in disadvantaged areas also showed positive short-term, but no long-term effects on weight development and reduced intake of unhealthy foods after providing individual parental support, including 1) health information, 2) motivational interviewing, and 3) teacher-led classroom activities with the children [44]. In the latter mentioned study, however, no intervention effects were shown on PA level [44]. Several studies have suggested that multi-component family-based behavioral interventions focusing on reducing sedentary time, increasing physical activity levels and decreasing fat and sugar in the diet in addition to parental involvement may be effective [45–47].

Collection of additional data from focus-group interview and semi-structured interviews provided valuable information about the challenges and attitudes among public health nurses and parents of overweight and obese children.

#### Results from the focus group interview of public health nurses

According to the national guidelines [6, 7], public health nurses are obliged to report childhood overweight and obesity to parents and provide further advice on weight management. The public health nurses participating in the focus group interview underlined the importance of addressing childhood overweight and obesity in a sensitive and respectful manner. However, all of the public health nurses, despite years of experience measuring children’s weight and height, found it difficult to initiate contact with parents and inform them about their child’s weight status. The public health nurses reported that many parents do not realize that their child is overweight or obese. Thus, conversations about weight status can be difficult for both public health nurses and parents. As a result of parents’ denial and resistance towards discussing weight issues related to their child, public health nurses were expressing needs to improve their own interpersonal communication skills. In addition, they also expressed a critical need for evidence based effective weight management programs, including practical tools supporting their efforts to promote healthy lifestyle habits in overweight and obese children and their families. Many of the public health nurses specifically described a need to improve their own nutritional knowledge and practical food-handling skills in order to intervene professionally with overweight/obese children and their parents. The need for increased nutritional knowledge among school nurses are supported by a report published by the Norwegian Directorate of Health [48].

#### Results from in depth-interviews of parents

Most of the parents being interviewed showed a positive attitude towards having healthy lifestyle habits. However, regulation challenges, high time pressure and having difficulties setting and maintaining healthy boundaries for their children were mentioned as the most important barriers to healthy lifestyle changes. By providing unhealthy snacks and sugar-sweetened beverages to their grandchildren, most of the parents also experienced that the children’s grandparents’ represented a barrier for adopting healthy eating habits. Half of the parents reported that their children were skipping breakfast every day due to decreased appetite in the morning. During the rest of the day, however, most of the parents described their children as always being hungry and constantly snacking. They also reported that their children showed taste preferences for food and beverages high in energy density (ED), which were commonly available in their home. Several of the parents also described that their overweight/obese child asked for large sized meals, and that they often, during main meals, consumed more food than their own parents. Most of the parents, however, found it difficult to assess appropriate portion sizes and establishing clear boundaries for what and how much their children are allowed to eat. In addition, many of the parents reported lack of regular family meals, high screen time, watching TV while eating meals and low levels of physical activity in everyday life.

#### Results from the pilot study

Experiences from the pilot study with overweight/obese children and their parents, confirmed the importance of having a community-based study weight management program, where locally based PHCs and HLCs are responsible for recruiting families and conducting the intervention, if possible in collaboration with locally based sports clubs. The health personnel responsible for recruiting families to the pilot study underlined the importance of addressing childhood overweight in a sensitive and respectful manner, and the health personnel in charge of organizing individual consultations at the local health clinic emphasized the importance of planning enough time (approximately 2 h) when conducting the first meeting. They experienced that the parents wanted to use this first meeting to discuss the risk of their child being stigmatized by entering the program, and elaborate their own weight and dieting experiences before identifying predictors and factors associated with the children’s overweight and/or obesity. Another important goal for this first meeting was to create an individual plan (IP) defining specific behavioral change goals in collaboration with health personnel. Despite the fact that some of the participating families were eager to set comprehensive goals, the health personnel experienced and emphasized the importance of defining a maximum of three main goals, followed by several more specific sub goals in the IP for each family during the intervention period. In order to support the families in reaching these goals, they were offered a minimum of three and a maximum of eight individual consultations during the intervention period. Motivational Interviewing (MI) was used as a counseling technique during all consultations to promote internal motivation, empowerment and mastery of health [49]. Both parents and health personnel responsible for the individual consultations reported that they were satisfied with the scope of the IP, the frequency of the individual consultations and the use of MI as a technique designed to change specific health behaviors.

Another part of the program involved participation of all the children in 60-min guided active play/physical activity twice a week, in which each participant was required to participate at least once a week. The trained instructors ensured focus on play based moderate- and vigorous intensity activities during these sessions. Additionally, parents were encouraged to plan and take part in fun physical activities together with their child, and suggestions of different locally based outdoor- and indoor activities were provided for each family. Both parents and children participating in this pilot study provided positive feedback related to participation in group-based physical activity sessions, and valued how all members of the family were motivated to engage in additional forms of physical sport activities together. Finally, the parents attended courses (four) providing information about the national nutrition recommendations and suggestions on how to increase intake of healthy food and beverages. An evaluation of the nutrition courses, however, indicated new courses tailored to meet the specific needs and challenges of this target group, should be developed. The revised courses should have both a theoretical and practical approach and involve both the overweight and obese children and their parents.

### Step 2: Development of change objective matrices

#### Program objective and outcomes

The needs assessment resulted in set of three key behaviors, physical activity, diet and regulation challenges, which were translated into ten program objectives (Table 1): 1) increase daily physical activity levels, 2) decrease sedentary time and screen viewing time, 3) establishing regular meal patterns, 4) increasing number of family meals, 5) regulate/decrease portion sizes, 6) increase intake of healthy food and beverages and decrease intake of unhealthy alternatives, 7) establish adequate sleep duration patterns, 8) increase self-esteem, 9) strengthen autonomy support (parents), and 10) strengthen autonomous regulation and self-efficacy (parents). In addition, two subsidiary program objectives were described: 1) reducing BMI-for age S-score (iso-BMI) and 2) improve self-perceived health.

**Table 1:** Specific program objectives of the intervention study

Key behaviors	Main objectives
ᅟPhysical activity	1. Increase daily physical activity levels
	2. Decrease sedentary time and screen viewing time
ᅟDiet	3. Establish regular meal patterns
	4. Increase number of family meals
	5. Regulate/decrease portion sizes
	6. Increase intake of healthy food and beverages and decrease intake of unhealthy alternatives
	*Sub-goals:* Lower the energy density of frequently consumed food and beverages: • Incorporate a large portion of fruits and vegetables into meals • Lean meats • Whole grains, legumes • Low-fat dairy products • Water instead of sugary beverages to quench thirst • Consume appetizers low in energy density
ᅟFamily structure	7. Establishing adequate sleep duration patterns
	8. Increase self-esteem
	9. Strengthening perceived autonomy support (parents)
	10. Strengthening autonomous regulation and self-efficacy (parents)
Health indicators	Subsidiary objectives
ᅟBMI/perceived health	1. Reduced BMI-for-age z-score (Iso-BMI)
	2. Improved self-perceived health

Performance objectives specify the behavioral action that the target groups have to perform in order to successfully change behavior. Because changes in lifestyle habits as part of a weight-management requires long-term self-management skills to regulate and adapt behavior to changing circumstances, self-regulation models were used to guide the creation of the performance objectives. An example of performance objectives is illustrated in Table 2.

**Table 2:** Performance objectives for improving healthy dietary habits and a meal patterns- an example

Self-regulation phase	Performance objectives
Monitoring phase	1. Families monitor current dietary habits and reflect on their own habits
Motivation phase	2. Families decide to participate in the program
	3. Families decide to challenge themselves and be actively involved in group sessions
Goal setting phase	4. Families set maximum three challenging but attainable goals to improve dietary habits
Active goal pursuit	5. Parents plan meal for weekdays including purchase of food and beverages according to plan
	6. Families have a regular meal pattern and decide to have one family meal every day
	7. Parents serve adequate portion size to their child
	8. Families select and try healthy alternatives to unhealthy food and beverages
	9. Families understand and resist temptation to increase intake of unhealthy food and beverages
	10. Parents buy and serve food healthy food and beverages regularly and reduce the availability of unhealthy alternatives
Evaluation phase	11. Families evaluate their achievements according to goals
	12. Families make long-term planning to maintain their newly acquired behavior

Further, important and changeable determinants for healthy lifestyle behaviors were selected. The personal determinants selected for increased physical activity and reduced sedentary time were 1) knowledge, 2) awareness, 3) attitude, 4) skills, 5) self-efficacy and 6) intention. In addition, social influence and availability (related to equipment and local facilities) were identified as important environmental determinants for physical activity and sedentary behavior.

The personal determinants selected for improving dietary habits were 1) knowledge, 2) awareness, 3) attitude, 4) skills and 5) self-efficacy. Social influence and availability of different food items and beverages were identified as important environmental determinants for dietary habits.

Change objectives were created by crossing the performance objectives with the selected determinants in matrices. An example of change objectives is illustrated in Table 3.

**Table 3:** Performance objectives related to changes in determinants with regard to improve dietary pattern- an example

	Personal					Home environment	
	Knowledge and risk-perception	Awareness	Attitude	Skills (self-regulation)	Self-efficacy	Social influence /reinforcement	Availability
Families select and try healthy alternatives to unhealthy food and beverages	1. Families are able to distinguish between healthy and unhealthy food and beverages 2. Families can describe the problems associated with increased intake of unhealthy snacks and drinks 3. Families can identify healthy alternatives to unhealthy snacks and drinks	1. Families can describe the health benefits of a high intake of healthy food and beverages (especially focus on increased intake of vegetables)	1. Families feel positive about making changes to increase intake of healthy food and beverages 2. Families express a positive attitude toward choosing healthy alternatives to unhealthy snacks and drinks	1. Families can explain to others the problems associated with a high intake of unhealthy food and beverages 2. Parents demonstrate setting appropriate limits for children regarding intake of unhealthy food and beverages 3. Families practices skills to ask for healthy alternatives in different settings 4. Families taste healthy alternatives to unhealthy snacks and drinks	1. Families express confidence in ability to recognize healthy and unhealthy food and beverages 2. Families show confidence to select healthy snacks and drinks 3. Families show confidence to try new snacks and drinks	1. Parents praises child for increasing healthy eating habits 2. Parents and other family members decrease intake of unhealthy food and beverages	1. Parents increases the availability and accessibility of healthy snacks and drinks at home 2. Parents reduces the availability and accessibility of unhealthy snacks and drinks at home

### Step 3: Selection of theory-based methods and strategies

#### Theoretical framework of the present study

To improve our understanding of how interventions effect change, we need to use precise and scientific methods for linking behavioral change theory to designing and evaluating interventions. Intervention descriptions are often not specific about the techniques employed and there is no clear correspondence between theoretical framework and adoption of particular change techniques [50, 51].

During the third step of the IM protocol, theory-based methods that were capable of changing the determinants were identified and chosen. An ecological approach was used when developing the intervention, which support the use of a multiple theories, as opposed to one single theory [31]. The present research program, however, used Self-Determination Theory (SDT) [52] as the main theoretical framework. Health-related behaviors are more likely to be initiated and maintained when the patients experience self-determination. This is done by maximizing the participants’ experience of the basic psychological needs of *autonomy*, *competence,* and *relatedness* in health care settings [53]. According to Basic Need Theory [54], a mini-theory within the Self-Determination Framework, the three innate needs must be satisfied for individuals to achieve positive development and optimal motivational function [53, 54]. The more autonomously regulated an individual is towards a given behavior, the greater effort, engagement, persistence, and stability the individual is likely to produce [55], and autonomous regulation has been identified as one of the key predictors of successful behavioral change [56]. The Norwegian Directory of Health [32] recommends MI as a counseling technique for the HLC’s. SDT may represent a theoretical approach to understand how MI works, and the two approaches may to some extent be integrated [57]. Meta-analysis of randomized controlled trials supports the effectiveness of MI in behavioral change and weight-loss treatment among adults [58]. MI supports the participants’ need for autonomy, relatedness and competence by allowing the participants freedom to explore reasons for and against change (autonomy) in a non-judgmental context (relatedness) and evoking changes that are realistic and attainable (competence) [55].

In addition to SDT, tools and materials used in the present study are also based on several other theoretical models, including the Health Believe Model, the Trans-Theoretical Model, the Precaution-Adoption Process Model, Persuation-Communication Matrix, Social Cognitive Theory, theory of Self-Regulation, Goal Setting Theory, Theory of Planned Behavior and Theories of Learning. The link between theoretical models, practical strategies, materials and tools are described in detail in Table 4.

**Table 4:** Description of methods and strategies used in the intervention study

Determinant	Theoretical methods	Strategy	Tools/materials
*1) Personal*			
Knowledge and risk perception (Diet, PA, Screen-time)	Consciousness raising (HBM; TTM)	Providing written and verbal information about the study	Letters sent to all parents of children in preschool and 3^rd^ grade providing information about the research study and target group Public health nurse (PHN) contact the target group (parents) by phone and invite them to participate in the study. An information folder, which explains participatory process to parents, is sent to their home-address or delivered during their first consult
		Provide tailored information to increase healthy lifestyle habits during the intervention period	Tailored brochures and information folders focusing on physical activity, diet, sleep and family structure
Awareness, risk perception & health believes (Diet, PA, Screen-time)	Information about personal risk (PAPM) Consciousness raising (HBM)	Personalized risk feedback from health screening	Expert registration/ monitoring and evaluation of BMI, dietary habits, physical activity level, sleep habits etc. in relation to national recommendations or recommendations by the research group
	Scenario-based risk information (PAPM)	Providing risk information	Tailored brochures focusing on long-term effects and information on benefits of healthy behavior
Attitude (Diet, PA, Screen-time)	Arguments (PCM) and feedback	Providing personal feedback to parents and children	PHN provides feedback on (perceived) positive consequences of healthy lifestyle habits Instructors give positive feedback during activity sessions
	Modeling (SCT)	Role models describing the benefits associated with a healthy lifestyle	Web movies
	Active learning (TSR)	Game promoting increased intake of fruit and vegetables	Rainbow shaped and colored vinyl sheets and colorful fruit and vegetable shaped self-adhesive stickers
Skills /self-regulation (PA) (Diet, PA, Screen-time)	Guided practice, active learning (SCT; TSR)	Organized activities with high intensity and different levels of coordination.	Tailored brochures and information folders describing how to organize different activities (for activity leaders) Information folders suggesting a variety of different high intensity exercises (for activity leaders)
		Provide specific suggestions for locally adapted parent-child leisure time activities (with high intensity) and strategies to reduce sedentary time/ screen time	Tailored brochures and oral information provided during individual family counselling meetings
		Parents and children prepare healthy meals together to improve hands-on practical cooking skills	A trained course coordinator. Detailed instructions describing necessary preparations and tips for conducting nutrition courses. Recipes for healthy dishes for all main meals
Self-efficacy (Diet, PA, Screen-time)	Goal setting and feedback (GST)	Formulation of maximum 3 challenging and feasible goals in close cooperation between health personnel and parents	PHN assist in goal setting using a contract that is signed by the parents
	Persuasive persuasion (SCT)	Encourage and convince parents to follow up the program	PHN-parents during individual family counselling meetings and power point presentations delivered as part of the course
	Guided practice, mastery experience, and feedback (SCT; TSR)	Organized activity groups (children) and cooking lessons (children and parents). Provide positive feedback whenever necessary and possible. Offer guided practice	Professional/trained instructors provide feedback during individual family counselling meetings and organized activity groups and cooking lessons
Intention (Diet, PA, Screen-time)	Autonomy building (SDT)	Motivational interviewing during individual coaching sessions	Trained PHN
	Persuasive communication (SCT)	Participation in group sessions, nutrition courses	Tailored educational material including power point presentations, verbal communication and information brochures
*2) Home environment*			
Social influence (Diet, PA, Screen-time)	Mobilize social support and resistance to social pressure (TPB)	Involve both parents, and significant others such as grandparents etc.	Tailored brochures and information folders and oral information provided during individual family counselling meetings
	Positive reinforcement (SCT)	Providing feedback evaluation of change process	The results from follow-up tests are delivered by PHN
Availability (Diet, PA)	Feedback, personal improvement, planning (TL; GST)	Practice in training, feedback on performance, and support with questions	Information folders and oral information provided during individual family counselling meetings and courses

*HBM* health believe model, *TTM* trans-theoretical model, *PAPM* precaution-adoption process model, *PCM* persuation-communication matrix, *SCT* social cognitive theory, *TSR* theory of self-regulation, *GST* goal setting theory, *SDT* self-determination theory, *TPB* theory of planned behavior, *TL* theories of learning

#### Strategies

The behavioral models and educational strategies are described in Table 4 and include individual family counselling meetings, nutritional courses, and physical activity groups providing general information, tailored information and practical learning sessions. Parents was at the same time encouraged to attend workshops intending to increase knowledge and awareness of regulation challenges, healthy eating habits and practical suggestions for implementing healthy behaviors into everyday life. Parents were educated on how to use these strategies at home, to further support their children in improving their behaviors. The intervention was developed in cooperation with the participating HLCs and PHCs, intending to comply with the national guidelines and being sustainable in everyday practice. A major strength of this community-based study is the support by the local leadership and collaboration with locally based sports clubs. The main outcomes and corresponding measurements are presented in Table 5. Most instruments are previously validated and have been used in the same age groups as intended in this study.

**Table 5:** Description of variables, purpose of measure, what we are to measure, relevant instruments, and when data will be collected

Ecological level	Purpose of measure	Variable	Measure	Instrument	When to collect
Child	SO, IC^a^	Dietary habits	Daily food intake, total energy-intake, meal pattern	FFQ^b^	At inclusion, 6, 12 and 24 months
	SO	Quality of life	Health-related quality of life, well-being	Kidscreen-10	At inclusion, 6, 12 and 24 months
	SO, IC	Motor control	Gross motor coordination	KTK Jumping Lateral^c^	At inclusion, 6, 12 and 24 months
	SO, IC	Physical activity /inactivity	Moderate-to-vigorous intensity physical activity	SenseWear Armband	At inclusion, 6, 12 and 24 months
	SO, IC	Physical activity	Self-efficacy	Described by Motl et al. [60]	At inclusion, 6, 12 and 24 months
	SO, IC	Physical activity	Enjoyment	Described by Motl et al. [60]	At inclusion, 6, 12 and 24 months
	SO, IC	Physical activity	Social support	Described by Sallis et al.	At inclusion, 6, 12 and 24 months
	SO, IC	Sleeping habits	Bedtime resistance, sleep duration, night wakings, daytime sleepiness	CSHQ^d^	At inclusion, 6, 12 and 24 months
	SO	Anthropometric measures	Weight and height (Iso-BMI)	Measured at the Child Health Centers	At inclusion, 6, 12 and 24 months
Parents	SO, IC	Motivation	Form of motivation	TSRQ^e^	At inclusion, 6, 12 and 24 months
	SO, IC	Perceived autonomy support	Support from health care providers	HCCQ 8^f^	6 and 12 months

^a^ *SO* study outcome, *IC* intervention component, ^b^ *FFQ* food frequency questionnaire, ^c^Körperkoordinationstest für Kinder, ^d^Childrens Sleep Habits Questionnaire, ^e^Treatment Self-Regulation Questionnaire, ^f^ Health Care Climate Questionnaire 8

### Step4: Creation of program components and materials

The program components, tools and materials were developed based on the results of the first three steps of the IM protocol and designed to increase healthy lifestyle habits, in particular by encouraging enjoying physical activity and healthy dietary habits, enhancing parental self-efficacy, enhance family engagement and parent-child relationships. The behavioral models and educational strategies include individual parental counselling and follow-up (3-8 meetings) using MI as a counselling method, nutritional courses (4-5 meetings) providing general knowledge and improving practical skills of nutrition for the whole family, and weekly physical activity sessions (1–2 times per week) for children aiming at increasing moderate-to-vigorous intensity physical activity (MVPA) and improving motor control.

Parents will be educated on how to use these strategies at home, to further support their children in improving their behaviors. The intervention material is developed in cooperation with the participating HLCs, intending to comply with the national guidelines and being sustainable in everyday life. Detailed information about program components and materials are presented in Table 4.

### Step5: Planning for adoption and implementation

The purpose of step 5 was to anticipate the adoption and implementation of the intervention study. To do so, representatives from Healthy Life Centre staff, municipality administration, county administration, general practitioners and representatives of three patient organizations were involved at an early stage of the intervention-development process [59]. Prior to conducting the intervention study, representatives of potential implementing organizations and the target group participated in focus-group interviews and in-depths interviews, respectively. This process identified facilitators and barriers for adoption and implementation, described under step 1. By participating in the pilot-study, representatives of the target group, health personnel and those responsible for activity groups helped to modify and improve the program.

#### Design of the study

**Fig. 1:**
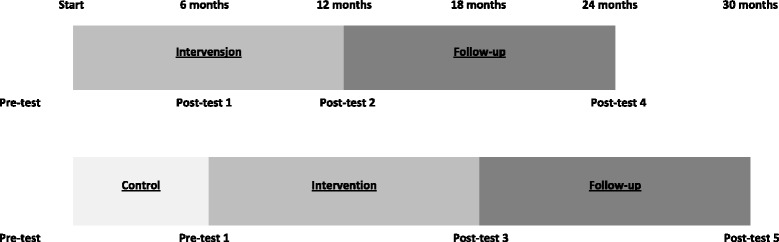
Study design describing pre- and post-testing

The present intervention study uses a quasi-experimental controlled design, as the intervention group and the control group are selected without any random pre-selection process. The control group will receive the same intervention as the intervention group with a 6-month delay. The study will investigate whether the intervention has a positive effect both at completion of the 6 months intervention and at long term follow up at 6 and 18 months after intervention period (Fig. 1). During different training sessions, health personnel responsible for recruitment and follow-up of participants have actively participated in developing their role in the implementation process, about the methods and strategies that they would use to facilitate behavioral change and how to use the tools and deliver the materials, which were tailored for the target group. Half-yearly meetings were also arranged where health personnel presented their own experiences and discussed strategies regarding adoption and implementation of the intervention study. In addition, all health personnel involved in the study received a handbook describing how the intervention should be delivered. They also received complete sets of course materials, tailored brochures and information folders etc., for distribution among all study participants (described in Table 4).

#### Population and power calculations

Families with children, 6–10 years old with an iso-BMI ≥ 25 will be invited to participate in the study. Follow up data from 64 participants in each group will give a statistical power of at least 80 % and a 5 % significance level for comparing effect measures of MVPA (difference 15 min, SD 30 min) between the groups. To account for the expected dropout and loss of follow-up rates of 25 %, assessing a higher dropout rate for the intervention group, we intend to include a minimum of 80 participants in both the intervention group and the control group.

### Step6: Development of an evaluation plan

The purpose of step 6 was the development of an evaluation plan. The main objectives specified in step 2 correspond with outcome variables and measurements, of which a detailed overview is described in Table 5. The primary outcome variables, measured at inclusion, at completion of the 6 months intervention and at long term follow up, are children’s MVPA, sedentary time, screen viewing time, eating habits, sleeping patterns and HRQoL as well as parental motivation and support from health care providers. The secondary outcome variables; iso-BMI of the children and parental perception of health will also be measured during pre- and post-testing described in Fig. 1.

In order to conduct a process-evaluation, important enhancers and barriers related to the adoption and implementation were identified by representatives (*n* = 3) involved in the adoption and implementation process in the pilot study. The in depth- interviews of important stakeholders identified the importance and value of having determined and highly committed initiators and project leaders, especially in the process of establishing agreements with the municipality council and other locally based partners. Barriers in the implementation process were related to poor cooperation between the program facilitators, municipal council and the health nurses. Resistance from the participating children’s parents and a lack of program funding were also identified as important barriers of adoption and implementation of the study.

## Discussion

This paper describes the developmental process and the content of a family-based intervention for improving lifestyle habits among overweight and obese children, 6–10 years old. Several studies have shown that a theory and evidence based approach increase the likelihood of positive intervention effects [30]. Despite a complex and time-consuming process, the IM protocol has been a useful tool for planning and development of the present intervention study.

Comprehensive systematic reviews highlight the successful use of multi-component interventions in the treatment of childhood obesity, and many studies have been conducted in clinical settings [29, 34, 60]. To our knowledge, only a limited number family-based, multi-component studies have been conducted in community-based settings in Norway. Thus, it was necessary to conduct a pilot study and obtain information on opportunities and barriers in order to tailor the intervention to the local conditions in Norwegian municipalities and to adapt the intervention tools and materials to health personnel conducting the study and to the target group.

Another strength of this process is the use of bottom-up approaches by including parents, public health nurses and other health personnel at Public Health Clinics (PHCs) and Healthy Life Centers (HLCs) in the planning process increases user-perspective and the likelihood of program sustainability long term. Although the interviews involved relatively few participants, they represented a broad specter of important key stakeholders influencing the weight-management program.

The research program has SDT as the main theoretical point of departure. Even though a logic model for understanding behavioral change is linear, focusing on the presumed cause-effect identified in theory and empirical research, we acknowledge that the behavioral treatment programs and proposed outcomes are part of a complex multilevel system, calling for an ecological approach. Targeting overweight and obese children and their parents is not an ultimate solution, as other environmental factors play pivotal roles that influence the children’s health-related behaviors. Parenting style and role modelling is, however, of crucial importance for children’s lifestyle behavior during this phase of life [61].

This intervention study will provide both objectively assessed data and self-reported data on both short-term and long-term effect of a multicomponent approach targeting overweight and obese children and their families. Furthermore, this study will provide practical and accessible tools and materials that can assist health care personnel in their daily practice. This program, if proven effective, may be implemented with contextual adaption in municipal health care institutions in Norway.
